# Electroacupuncture alleviates mechanical allodynia and anxiety‐like behaviors induced by chronic neuropathic pain via regulating rostral anterior cingulate cortex‐dorsal raphe nucleus neural circuit

**DOI:** 10.1111/cns.14328

**Published:** 2023-07-03

**Authors:** Yingling Xu, Xixiao Zhu, Yuerong Chen, Yeqing Chen, Yichen Zhu, Siqi Xiao, Mengwei Wu, Yifang Wang, Chi Zhang, Zenmin Wu, Xiaofen He, Boyu Liu, Zui Shen, Xiaomei Shao, Jianqiao Fang

**Affiliations:** ^1^ Key Laboratory of Acupuncture and Neurology of Zhejiang Province The Third Clinical Medical College, Zhejiang Chinese Medical University Hangzhou China; ^2^ NHC and CAMS Key Laboratory of Medical Neurobiology, MOE Frontier Science Center for Brain Research and Brain‐Machine Integration, School of Brain Science and Brain Medicine Zhejiang University Hangzhou China; ^3^ Liangzhu Laboratory Zhejiang University Medical Center Hangzhou China

**Keywords:** anxiety‐like behaviors, chronic neuropathic pain, dorsal raphe nucleus, electroacupuncture, neural circuit, rostral anterior cingulate cortex

## Abstract

**Aims:**

Epidemiological studies in patients with neuropathic pain have demonstrated a strong association between neuropathic pain and psychiatric conditions such as anxiety. Preclinical and clinical work has demonstrated that electroacupuncture (EA) effectively alleviates anxiety‐like behaviors induced by chronic neuropathic pain. In this study, a potential neural circuitry underlying the therapeutic action of EA was investigated.

**Methods:**

The effects of EA stimulation on mechanical allodynia and anxiety‐like behaviors in animal models of spared nerve injury (SNI) were examined. EA plus chemogenetic manipulation of glutamatergic (Glu) neurons projecting from the rostral anterior cingulate cortex (rACC^Glu^) to the dorsal raphe nucleus (DRN) was used to explore the changes of mechanical allodynia and anxiety‐like behaviors in SNI mice.

**Results:**

Electroacupuncture significantly alleviated both mechanical allodynia and anxiety‐like behaviors with increased activities of glutamatergic neurons in the rACC and serotoninergic neurons in the DRN. Chemogenetic activation of the rACC^Glu^‐DRN projections attenuated both mechanical allodynia and anxiety‐like behaviors in mice at day 14 after SNI. Chemogenetic inhibition of the rACC^Glu^‐DRN pathway did not induce mechanical allodynia and anxiety‐like behaviors under physiological conditions, but inhibiting this pathway produced anxiety‐like behaviors in mice at day 7 after SNI; this effect was reversed by EA. EA plus activation of the rACC^Glu^‐DRN circuit did not produce a synergistic effect on mechanical allodynia and anxiety‐like behaviors. The analgesic and anxiolytic effects of EA could be blocked by inhibiting the rACC^Glu^‐DRN pathway.

**Conclusions:**

The role of rACC^Glu^‐DRN circuit may be different during the progression of chronic neuropathic pain and these changes may be related to the serotoninergic neurons in the DRN. These findings describe a novel rACC^Glu^‐DRN pathway through which EA exerts analgesic and anxiolytic effects in SNI mice exhibiting anxiety‐like behaviors.

## Graphical abstract

The rACC^Glu^‐DRN circuit works under the pathological state but not under the physiological state, and plays an important role in regulating anxiety‐like behaviors related to neuropathic pain as neuropathy develops. Electroacupuncture exerts analgesic and anxiolytic effects on chronic neuropathic pain and related anxiety through the rACC^Glu^‐DRN pathway.

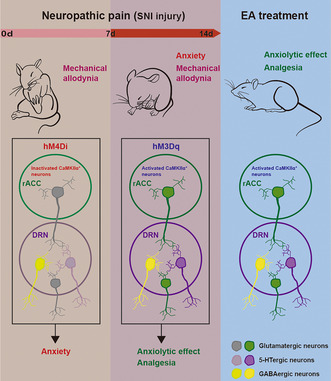

## 1 INTRODUCTION

Chronic neuropathic pain, defined as nervous system lesions or diseases associated with persistent or recurrent pain for more than 3 months, affects up to 10% of the general population.^1^ Clinical anxiety is highly prevalent in patients with peripheral neuropathic pain.^2^, ^3^ Currently recommended first‐line treatments for neuropathic pain include anti‐depressants and anti‐convulsants,^1^ which both have obvious side effects. The lack of effective strategies for treating chronic neuropathic pain (especially cases accompanied by emotional disorders) may be due to a lack of clarity regarding its underlying mechanisms.

The rostral anterior cingulate cortex (rACC) is a key structure that mediates the negative affective component of chronic pain.^4^ Glutamatergic neurons in the rACC are necessary and sufficient to induce pain‐related negative emotions.^5^, ^6^ Our previous studies have demonstrated that glutamatergic neurons in the rACC (rACC^Glu^) serve as part of the neural circuitry underlying anxiety‐like behaviors induced by chronic pain.^7^, ^8^ We hypothesized that glutamatergic transmission in the rACC might play a crucial role in processing the anxiety‐related aspects of chronic neuropathic pain. Therefore, in the present study, we focused on the role of rACC^Glu^ neurons as a descending pain‐modulating pathway in regulating anxiety related to chronic neuropathic pain.

The dorsal raphe nucleus (DRN) contains approximately one‐third of all serotoninergic neurons in the brain.^9^ Serotonin (5‐HT) is involved in pain inhibition, and 5‐HT antagonists block the analgesic effect of some anti‐depressants.^10^ Studies^11^, ^12^, ^13^ have indicated that the DRN 5‐HTergic system is involved in the comorbidity of chronic neuropathic pain and emotional disorders. The activity of 5‐HTergic neurons alters as neuropathic pain progresses, and reduced activity of 5‐HTergic neurons is linked to the emergence of anxiety.^13^ The DRN may play an important role in emotional disorders associated with chronic pain through connecting with other brain regions.^14^ Weissbourd et al.^15^ have found that 5‐HTergic neurons in the DRN receive synaptic inputs from glutamatergic neurons in the anterior neocortex, indicating the presence of a connection between the rACC and DRN. At present, it remains unknown whether the rACC^Glu^‐DRN circuit is involved in the comorbidity of chronic neuropathic pain and anxiety.

Recent clinical evidence has demonstrated that electroacupuncture (EA) effectively relieves various types of pain disorders.^16^, ^17^, ^18^ Clinically, EA significantly improves anxiety associated with pain in breast cancer patients.^19^ Basic research has suggested that EA effectively relieves mechanical hypersensitivity^20^, ^21^ and anxiety‐like behaviors^22^ under chronic neuropathic pain, indicating that EA may play an important role in chronic neuropathic pain. EA performed at the “Zusanli” (ST36) and “Sanyinjiao” (SP6) points produces analgesic or anxiolytic effects in cases of inflammatory pain^23^, ^24^ and neuropathic pain.^25^ The analgesic and anti‐depressive effects of EA at ST36 and SP6 on the pain‐depression dyad are mediated by 5‐HT in the DRN.^26^ Additionally, EA ameliorated pain sensation through the rACC^Glu^‐vlPAG circuit without relieving anxiety‐like behaviors in SNI mice,^8^ indicating that the circuitry underlying EA's analgesic and anxiolytic effects needs further investigation in the context of comorbid chronic neuropathic pain and anxiety.

In this study, we explored the functional organization of the rACC^Glu^‐DRN circuit and investigated the effect of this novel circuit in a male mouse model of spared nerve injury (SNI). Furthermore, we combined chemogenetic methods and EA treatment to assess whether the circuit identified in the SNI model was relevant to the analgesic and anxiolytic effects of EA. Based on our findings, we propose that the EA‐induced alleviation of mechanical allodynia and anxiety‐like behaviors is associated with the modulation of the rACC^Glu^‐DRN circuit in the SNI mouse model.

## 2 METHODS

### 2.1 Animals

All experiments used adult male C57BL/6J mice at 8–10 weeks of age. These mice were housed in the Laboratory Animal Center of Zhejiang Chinese Medical University accredited by the Association for Assessment and Accreditation of Laboratory Animal Care (AAALAC). The mice were housed five per cage in a colony and provided with ad libitum access to water and food (standard mouse chow). They were maintained under a 12‐h light/dark cycle at a consistent temperature (23–25°C). Mice were randomly assigned to experimental groups. The mice were given a minimum of 1 week to adapt to the new environment before the experiment. All animal protocols were approved by the Animal Care and Use Committee of Zhejiang Chinese Medical University (Permission Number: IACUC‐20190225‐01). All animals were used in the various experimental designs as shown in Table S1 in the Appendix S1.

### 2.2 Experimental designs

**FIGURE 1:**
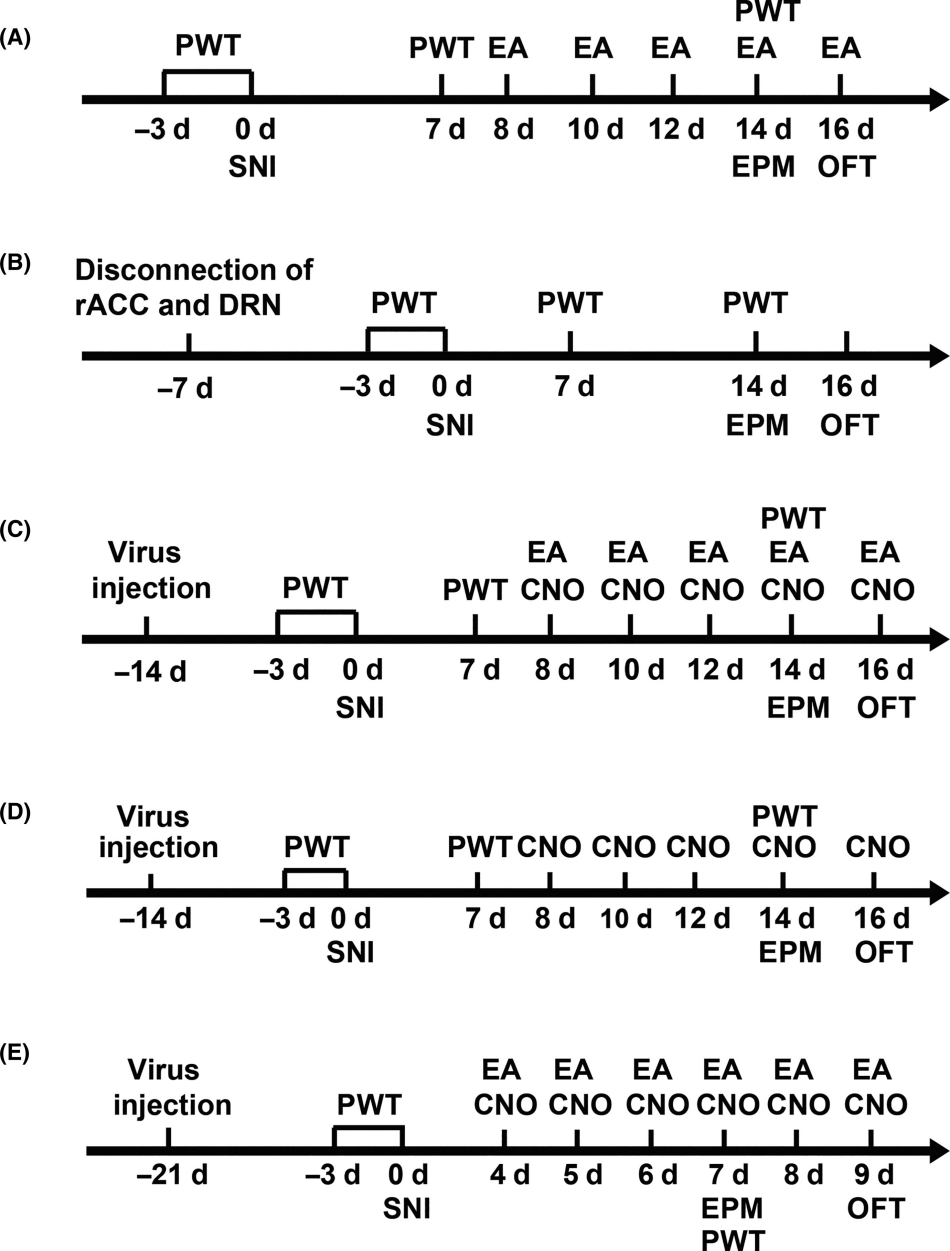
Timeline of different experimental designs. (A) Timeline of the SNI surgery, electroacupuncture (EA), and behavioral testing to study the analgesic and anxiolytic effects of EA treatment in SNI 14d mice. (B) Timeline for disconnection of rACC and DRN circuit in regulating mechanical allodynia and anxiety‐like behaviors under SNI. (C) Timeline for viral injection, CNO treatment, EA, and behavioral testing to study the role of this circuit in mechanical allodynia and anxiety‐like behaviors in SNI 14d mice and the intervention of EA. (D) Timeline for viral injection, CNO treatment, and behavioral testing to study the role of inhibiting this circuit in naïve mice. (E) Timeline for exploring the role of inhibiting rACC^Glu^‐DRN circuit in SNI 7d mice and the effects of EA.

To study the effect of EA treatment on mechanical allodynia and anxiety‐like behaviors related to SNI mice, EA was performed at 8, 10, 12, 14, and 16 days after SNI as shown in Figure 1A. The PWT was tested before SNI and at 7 and 14 days after SNI. The EPM was performed on day 14 and the OFT was tested on day 16 after SNI.

To demonstrate the role of disconnection of rACC and DRN circuit in regulating mechanical allodynia and the anxiety‐like behaviors under SNI, the experimental design is shown in Figure 1B. The connection between rACC and DRN was disrupted at 7 days before the SNI surgery.

As shown in Figure 1C, chemogenetic manipulation (activation or inhibition) of rACC‐DRN circuit combined with EA treatment was used to study the role of this circuit in mechanical allodynia and anxiety‐like behaviors in SNI 14d mice and the intervention of EA. The virus was injected at 14 days before the SNI surgery. EA was performed 30 min after Clozapine‐N‐oxide (CNO, i.p., BrainVTA) at 8, 10, 12, 14, and 16 days post‐SNI.

As shown in Figure 1D, chemogenetic inhibition of rACC‐DRN circuit was used to study the role of this circuit in mechanical allodynia and anxiety‐like behaviors in naïve mice. The behavioral tests were performed 30 min after CNO.

To further clarify the role of the circuit, chemogenetic inhibition of rACC‐DRN circuit combined with EA treatment was used in SNI 7d mice as shown in Figure 1E. The virus was injected at 21 days before the SNI surgery. EA was performed 30 min after CNO at 4, 5, 6, 7, 8, and 9 days post‐SNI. The PWT was tested before SNI and at 7 days after SNI. The EPM was performed at 7 days and the OFT was tested at 9 days after SNI.

### 2.3 Animal model of neuropathic pain

**FIGURE 2:**
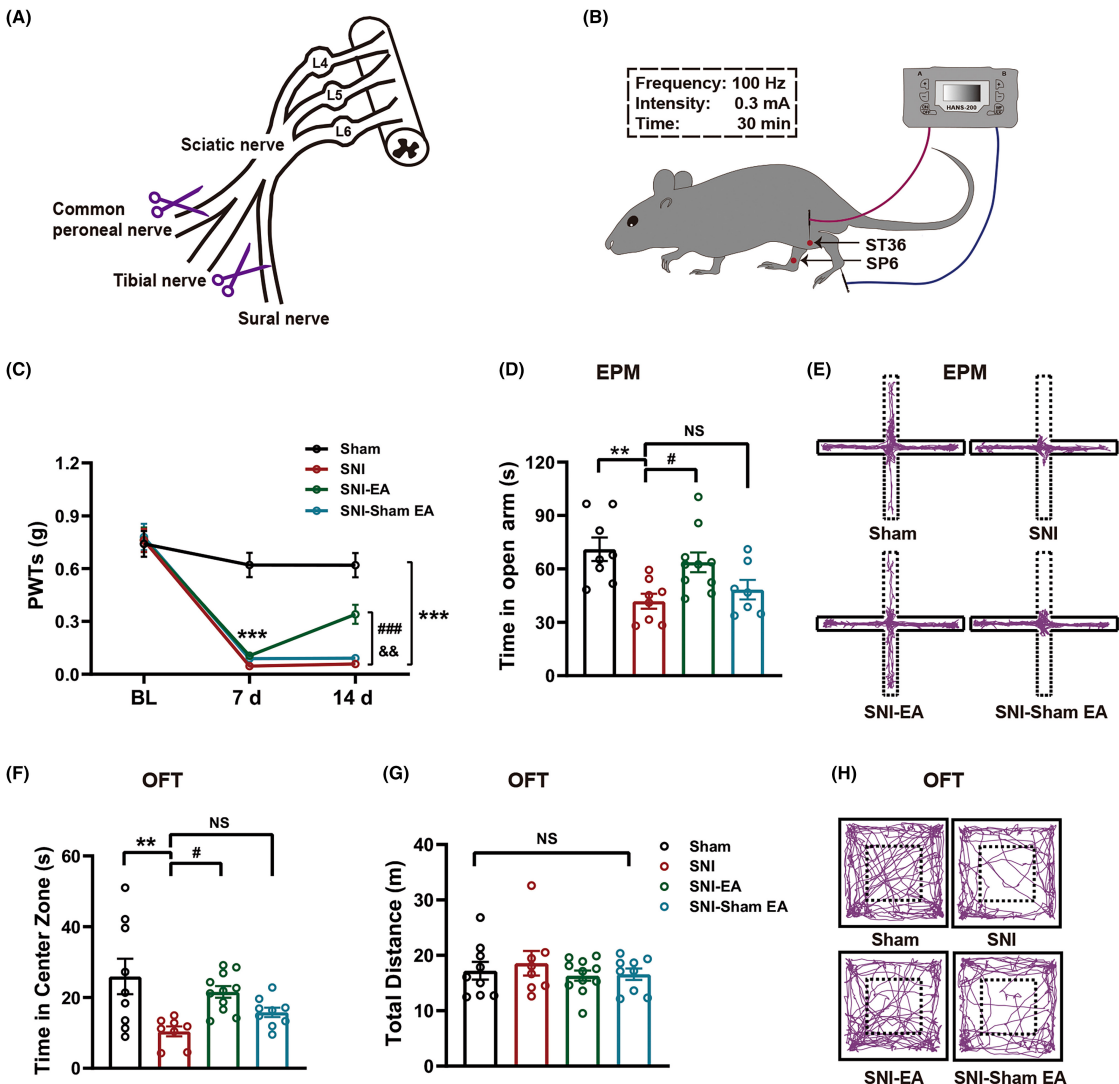
Electroacupuncture (EA) exerted analgesic and anxiolytic effects in SNI mice. (A) Diagram of the sciatic nerve and its terminal branches, including manipulations performed to construct the SNI model of neuropathic pain. (B) Schematic of EA at the ST36 and SP6 sites. (C) Time course of EA on pain sensory of SNI mice (*n* = 10–11). (D) EA increased time in open arms decreased by SNI in the EPM (*n* = 7–10). (E) Representative animal tracks of the four groups in the EPM. (F,G) EA upregulated time in central area reduced by SNI in the OFT (F) (*n* = 8–11) without affecting locomotor activity (G). (H) Representative animal tracks of the four groups in the OFT. Data are expressed as the mean ± SEM. **p < 0.01 and ***p < 0.001 compared with sham mice, ^#^ *p* < 0.05 and ^###^ *p* < 0.001 compared with SNI mice, ^&&^ *p* < 0.01 compared with SNI‐sham EA mice. NS, not significant.

All mice were deeply anesthetized with 2% isoflurane during the surgery. For the SNI group, the left thigh and muscle were incised and gently separated to expose the sciatic nerve, which consists of three terminal branches (the sural, common peroneal, and tibial nerves). As shown in Figure 2A, the common peroneal and sural nerves were tightly ligated with 6–0 silk sutures. To prevent reattachment, the ligated branches were transected distal to the ligature, and approximately 2 mm of each distal nerve stump was removed, leaving only the tibial nerve intact.^27^, ^28^ The overlying muscle and skin layers were sutured and disinfected with iodophor. In the sham for SNI model, sham mice were subjected to the same operation, but the nerves were left intact.

### 2.4 Disconnection of rACC and DRN circuit

For disconnection of rACC and DRN circuit, the mice were positioned in a stereotactic frame (68,025, RWD) under 0.3% pentobarbital sodium (30 mg/kg, i.p.) analgesia. A heating pad was used to maintain mouse's body temperature at 37°C. The coordinates are listed below (in mm): dorsoventral (DV) from the brain surface, anterior‐posterior (AP) from bregma, and mediolateral (ML) from the midline.

After craniotomy, the mice were injected with drugs using calibrated glass microelectrodes connected to an infusion pump (micro 4, WPI). Mice were microinjected with the excitotoxin quinolinic acid (QA, 90 mM, in the 0.9% saline, pH = 7.4, P63204, Sigma)^29^ into the unilateral rACC (AP, +1.34 mm; ML, −0.25 mm; DV, −0.85 mm) and 5,7‐dihydroxytryptamine (5,7‐DHT, 52 mM, in 0.1% ascorbic acid, SML2058, Sigma) into the contralateral DRN (AP, −4.39 mm; ML, +0.05 mm; DV, −2.69 mm) to bilaterally disrupt interhemispheric neural communication between the rACC and DRN (Figure 4E). The rate of infusion was 60 nL/min, and the total volume was 250 nL for the rACC and 300 nL for the DRN. The needle was left in place for an additional 10 min to ensure reagent diffusion. One week after the surgery for disconnection of rACC and DRN circuit, the distal nerve stump of the common peroneal and sural nerves was removed. The timeline of the experimental design is shown in Figure 1B.

### 2.5 Viral injection

For anterograde tracing of the rACC‐DRN circuit, we injected AAV2/9‐CaMKIIα‐EGFP (5.24 × 10^12^ vg/mL, 80 nL, PT‐0290) into the right rACC (AP, +1.32 mm; ML, −0.25 mm; DV, −0.85 mm) at a rate of 60 nL/min in naïve mice. For retrograde monosynaptic tracing, AAV2/R‐CaMKIIα‐EGFP (5.34 × 10^12^ vg/mL, 150 nL, PT‐0290) was injected into the DRN (AP, −4.39 mm; ML, −0 mm; DV, −2.69 mm) at a rate of 80 nL/min in naïve mice. After injection, the pipette was retained in the injection site for an additional 8‐10 min to facilitate the diffusion of the virus and then slowly withdrawn. The mice were moved to their home cages after full recovery from anesthesia in a 37°C environment. Three weeks after the tracing viral infection, the mice were anesthetized and sacrificed for collecting brain samples.

rAAV2/9‐CaMKIIα‐DIO‐hM3Dq‐mCherry‐WPRE‐pA (3.04 × 10^12^ vg/mL, 80 nL, PT‐1144, unilaterally injected into the right rACC) and rAAV2/9‐CaMKIIα‐DIO‐hM4Di‐mCherry‐WPRE‐pA (3.38 × 10^12^ vg/mL, 80 nL, PT‐1143, bilaterally injected into the rACC) vectors were used for chemogenetic manipulations. rAAV2/9‐CaMKIIα‐DIO‐mCherry‐WPRE‐pA (5.35 × 10^12^ vg/mL, 80 nL, PT‐1167) vectors were used as the control. rAAV2/R‐CaMKIIα‐Cre (6.65 × 10^12^ vg/mL, 150 nL, PT‐0220) vector was injected into the DRN. All viruses were purchased from BrainVTA. For chemogenetic activation of the neurons projecting from the rACC to the DRN, CNO (1 mg/mL, dissolved in DMSO and diluted in 0.9% saline, 10 mg/kg, i.p.) was administered 30 min before the behavioral tests.^30^

### 2.6 Electroacupuncture (EA) treatment

As shown in Figure 2B, EA was performed with continuous‐mode stimulation for 30 min, with an electrical current of 0.3 mA and a frequency of 100 Hz using a HANS Acupoint Nerve Stimulator (HANS‐200A, Beijing Hua Wei Industrial Development Co). EA was bilaterally performed at the ST36 (Zusanli; located 2 mm lateral to the anterior tubercle of the tibia in the anterior tibial muscle and 4 mm distal to the knee joint lower point) and the SP6 (Sanyinjiao; located approximately 3–5 mm above the tip of the medial malleolus and at the posterior border of the tibia) acupoints by inserting the 0.16 × 7‐mm unipolar stainless steel acupuncture needle electrode approximately 2–3 mm deep at each site.^31^, ^32^, ^33^, ^34^ The sham and SNI groups were loosely immobilized without EA. Sham EA was performed by inserting acupuncture needles into the ST36 and SP6 acupoints without applying any current; this group served as a negative control.

### 2.7 Paw withdrawal threshold

Mechanical allodynia was assessed by measuring the 50% paw withdrawal threshold (PWT) in response to von Frey monofilaments (UGO). The mice were placed in a plastic cage. Each mouse was allowed to acclimate to the observation chamber (7 × 9 × 10 cm) on top of a mesh grid for approximately 60 min prior to testing. Then, von Frey filaments were used in ascending order from 0.04 to 1.4 g to stimulate the plantar surface of the left hind paw. The mechanical threshold was defined as the force that elicited a rapid withdrawal response at least three times out of five repeated stimuli.^35^

### 2.8 Assessment of anxiety‐like behaviors

Anxiety‐like behaviors of mice were recorded and analyzed in the elevated plus maze (EPM) test and open‐field test (OFT).^8^ The timeline for anxiety‐like behavioral tests is shown in Figure 1.

#### 2.8.1 Elevated plus maze test

The EPM consisted of a central platform (6 × 6 cm) with two opposite closed arms (30 × 6 × 15 cm) and two opposite open arms (30 × 6 cm), creating a plus shape. The plus‐shaped platform was 40 cm above the floor in a silent and dimly lit room. Mice were individually placed on the central platform facing an open arm and were allowed to explore the maze for 5 min. Mouse behavior was recorded with a camera. The time spent in the open arms was measured to evaluate anxiety using ANY‐maze (Stoelting Co.). The maze was cleaned with 75% ethanol between tests.

#### 2.8.2 Open‐field test

Mice were gently placed at the center of a cubic chamber (40 × 40 × 40 cm) at the beginning of the assay and allowed to freely explore for 5 min. Their movement was recorded and analyzed with ANY‐maze (Stoelting Co.). The time spent in the center of the chamber (20 × 20 cm) represented the anxiety of mice, and the total distance traveled indicated the locomotor activity. All mice were habituated to the testing room for 12 h before the OFT. The testing room was dimly illuminated with indirect white lighting. The open‐field chamber was cleaned with 75% ethanol after each test to remove olfactory cues from the apparatus.

### 2.9 Immunohistochemistry

The mice were deeply anesthetized with pentobarbital sodium (30 mg/kg, i.p.) about 90 min after the OFT and sequentially perfused through the ascending aorta with 0.9% saline followed by 4% (w/v) paraformaldehyde. After perfusion, mouse brains were removed and post‐fixed in 4% paraformaldehyde at 4°C for 24 h. After cryoprotection of the brains with 15% and 30% (w/v) sucrose, coronal sections (20 μm thick) were cut on a cryostat (CryoStar NX50, Thermo Fisher) and used for immunofluorescence. The sections were blocked with 10% donkey serum (with 0.3% Triton X‐100) for 1 h at 37°C and incubated at 4°C overnight with primary antibodies diluted in blocking buffer, including anti‐c‐Fos (1:800, rabbit, Abcam, ab190289), anti‐CaMKIIα (1:200, rabbit, Abcam, ab5683), anti‐serotonin (1:1000, rabbit, ImmunoStar, 20,080), a mixture of anti‐CaMKIIα (1:200, mouse, Abcam, ab22609) and anti‐c‐Fos (1:800, rabbit, Abcam, ab190289), and a mixture of anti‐serotonin (1:1000, goat, Abcam, ab66047) and anti‐c‐Fos (1:800, rabbit, Abcam, ab190289). Subsequently, the sections were incubated with the corresponding fluorophore‐conjugated secondary antibodies (1:800) for 1 h at 37°C. Fluorescence signals were visualized using a virtual slide microscope (VS120‐S6‐W, Olympus).

### 2.10 Nissl staining

After the OFT at 16 days post‐SNI, the mice were sacrificed and perfused with sterile saline followed by 4% formaldehyde. Brains were cryoprotected and sectioned into 30‐μm‐thick slices for Nissl staining. According to the manufacturer's protocol, Nissl Staining Solution (Beyotime) was evenly added to the slices; these slices were stained for 3–10 min, washed with double‐distilled H_2_0 (ddH_2_O) twice (each time for several seconds), then washed with 95% ethanol for approximately 5 s, and finally washed with 70% ethanol. After drying, the slices were observed by an Olympus VS120‐S6‐W microscope.

### 2.11 Statistical analysis

Statistical analysis was performed using GraphPad Prism. Shapiro‐Wilk test was applied to check the normal distribution of the data, while *F*‐test was used to check homogeneity of variances. Data were presented as mean ± SEM (*n* ≥ 5), and were compared between groups using two‐tailed *t*‐test or one‐way ANOVA followed by Tukey's test when normally distributed. Mann‐Whitney U and Kruskal‐Wilcoxon tests were performed if the data were not normally distributed. *p* < 0.05 was considered statistically significant.

## 3 RESULTS

### 3.1 EA significantly alleviated both mechanical allodynia and anxiety‐like behaviors in a SNI mouse model

We established a mouse model of chronic peripheral neuropathic pain via SNI following previously reported protocols^27^, ^28^ (Figure 2A). The timeline is shown in Figure 1A. The SNI mice displayed obvious signs of mechanical allodynia in the ipsilateral hind limbs, as measured by a significant reduction in the PWT (Figure 2C). Mechanical allodynia in the SNI mice could persist at least for 14 days after SNI, consistent with previous results.^8^, ^25^ Anxiety‐like behaviors have been widely reported in the SNI mouse model.^36^ The EPM and OFT are commonly used to assess anxiety‐related behaviors.^37^, ^38^ Fourteen days after SNI, the mice displayed anxiety‐like behaviors in the EPM (i.e., reduced time in the open arms; Figure 2D) and the OFT (i.e., reduced time in the center zone; Figure 2F). Notably, the total distance traveled in the OFT did not significantly differ between the SNI 14d mice and the sham mice (Figure 2G). These results suggest that anxiety‐like behaviors are reliably induced by the model of chronic neuropathic pain.

We then examined the effects of EA on mechanical allodynia and anxiety‐like behaviors in SNI mice. As illustrated in Figure 2B, we administered 30 min of EA to the bilateral ST36 and SP6 acupoints located in the hind limbs of the mice every other day beginning on the 8th day after SNI, with current intensity of 0.3 mA and frequency of 100 Hz. EA significantly increased the PWT (Figure 2C) and the time spent in the open arms of the EPM (Figure 2D) on day 14, and mice treated with EA spent more time in the center of the OFT on day 16 (Figure 2F); these findings indicate that EA induced anti‐hyperalgesic and anxiolytic effects in SNI mice. SNI mice with sham EA did not exhibit reduced mechanical allodynia (Figure 2C) and anxiety‐like behaviors (Figure 2D,F). Representative animal tracks in the EPM and OFT are shown in Figure 2E,H, respectively. These data suggest that EA significantly attenuates mechanical allodynia and anxiety‐like behaviors in the SNI mouse model.

### 3.2 SNI‐induced decreases in the activity of glutamatergic neurons in the rACC and 5‐HTergic neurons in the DRN were alleviated by EA

**FIGURE 3:**
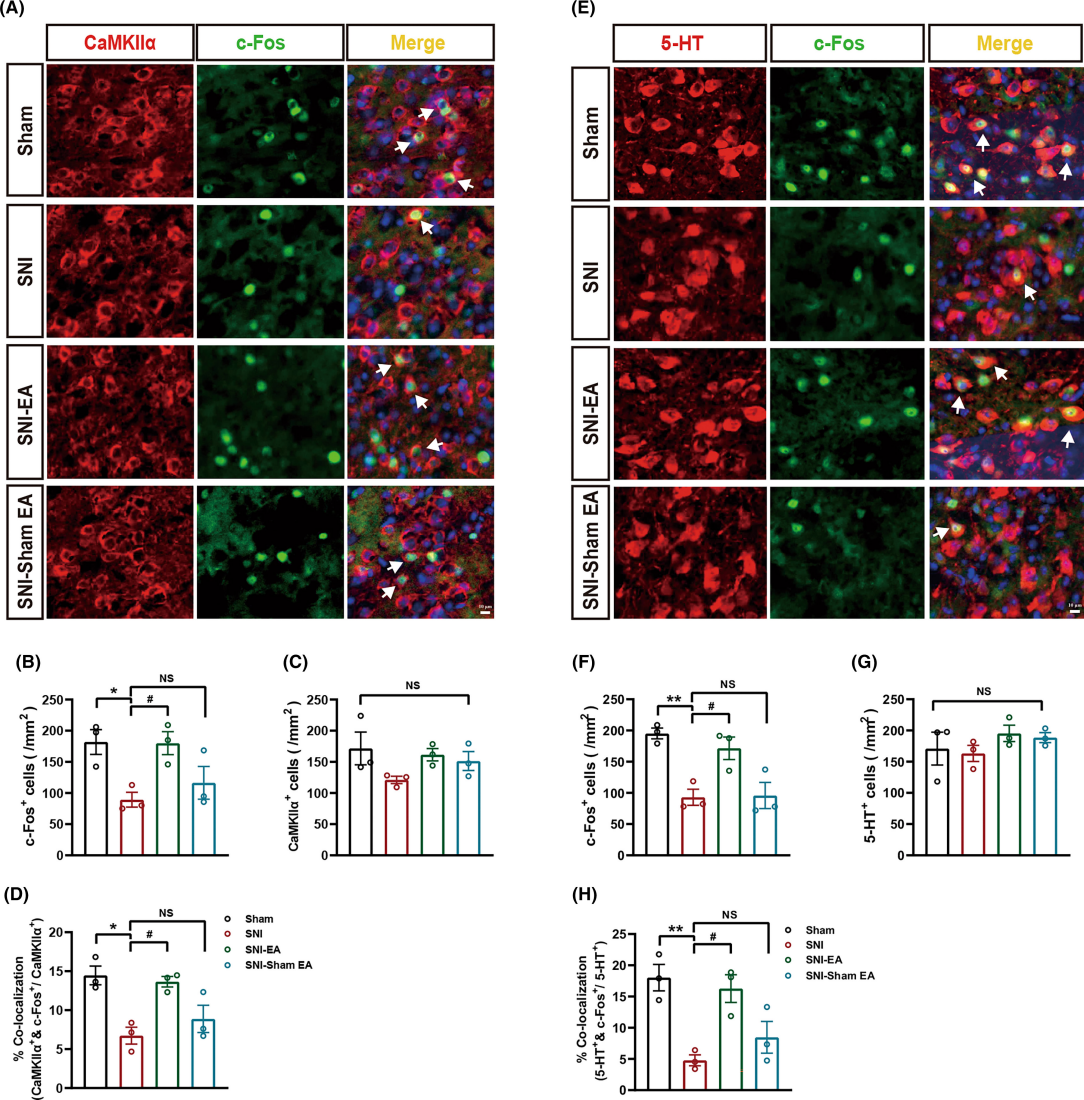
Electroacupuncture (EA) activated glutamatergic neurons in the rACC and serotonergic neurons in the DRN of SNI mice exhibiting anxiety‐like behaviors. (A) Representative images indicating that CaMKIIα (red) co‐stained with c‐Fos (green) in the rACC from the sham, SNI, SNI‐EA, and SNI‐sham EA groups. Arrows indicate c‐Fos expression in CaMKIIα^+^ neurons; scale bar, 10 μm. (B) The number of c‐Fos‐positive neurons in the rACC was significantly increased after EA (*n* = 3). (C) The number of CaMKIIα‐positive neurons in the rACC did not change (*n* = 3). (D) Proportion of neurons expressing CaMKIIα that were co‐labeled with c‐Fos in the rACC (*n* = 3). (E) Representative images indicating that 5‐HT (red) co‐stained with c‐Fos (green) in the DRN of the sham, SNI, SNI‐EA, and SNI‐sham EA groups. Arrows indicate c‐Fos expression in 5‐HT^+^ neurons; scale bar, 10 μm. (F) The number of c‐Fos‐positive neurons in the DRN was significantly increased by EA (*n* = 3). (G) The number of 5‐HT‐positive neurons in the DRN did not change (*n* = 3). (H) Proportion of neurons expressing 5‐HT co‐labeled with c‐Fos in the DRN (*n* = 3). At least five slices were taken from each mouse. Data are expressed as the mean ± SEM. *p < 0.05 and **p < 0.01 compared with sham mice, ^#^p < 0.05 compared with SNI mice. NS, not significant.

The rACC^8^, ^39^ and the DRN^13^, ^14^ are both key regions for comorbid chronic neuropathic pain and affective disorders. rACC^Glu^ neurons play a critical role in regulating anxiety‐like behaviors during chronic pain, as shown by our previous studies.^7^, ^8^ DRN^5‐HT^ neurons serve as a possible substrate for the affective component of chronic neuropathic pain.^12^, ^13^, ^40^ Therefore, we next explored whether EA‐induced anti‐allodynic and anxiolytic effects in SNI mice were related to rACC^Glu^ and DRN^5‐HT^ neurons. Representative images of CaMKIIα (red) co‐stained with c‐Fos (green) in the rACC at day 16 post‐surgery as well as representative images of 5‐HT (red) co‐stained with c‐Fos (green) in the DRN shown in Figure 3A,E, respectively. Immunostaining indicated that the number of c‐Fos (a widely used indicator of cell activation)‐positive neurons was significantly decreased in the rACC (Figure 3B) as well as in the DRN (Figure 3F) compared with the sham group, suggesting that the rACC and DRN are both involved in comorbidities of chronic neuropathic pain and anxiety‐like behaviors without influencing the total CaMKIIα^+^ neurons (Figure 3C) or 5‐HT^+^ neurons (Figure 3G). As shown in Figure 3D, compared with sham mice, SNI mice had fewer CaMKIIα‐expressing neurons co‐labeled with c‐Fos in the rACC. Similarly, compared with sham mice, 5‐HT‐expressing neurons co‐labeled with c‐Fos in the DRN were reduced in SNI mice (Figure 3H). After EA, c‐Fos‐positive neurons in the rACC (Figure 3B) and DRN (Figure 3F) were both increased without changing the total number of CaMKIIα^+^ neurons (Figure 3C) or 5‐HT^+^ neurons (Figure 3G). EA also upregulated the activity of CaMKIIα neurons in the rACC (Figure 3D) and the activity of 5‐HTergic neurons in the DRN (Figure 3H). In SNI mice, sham EA did not change the activities of CaMKIIα neurons (Figure 3D) and 5‐HTergic neurons (Figure 3H). These data indicate that the activities of CaMKIIα neurons in the rACC and 5‐HTergic neurons in the DRN are both decreased under chronic neuropathic pain comorbidities with anxiety‐like behaviors; EA effectively reverses these changes.

### 3.3 Neuronal projections between the rACC and DRN were required for SNI‐induced mechanical allodynia and anxiety‐like behaviors

**FIGURE 4:**
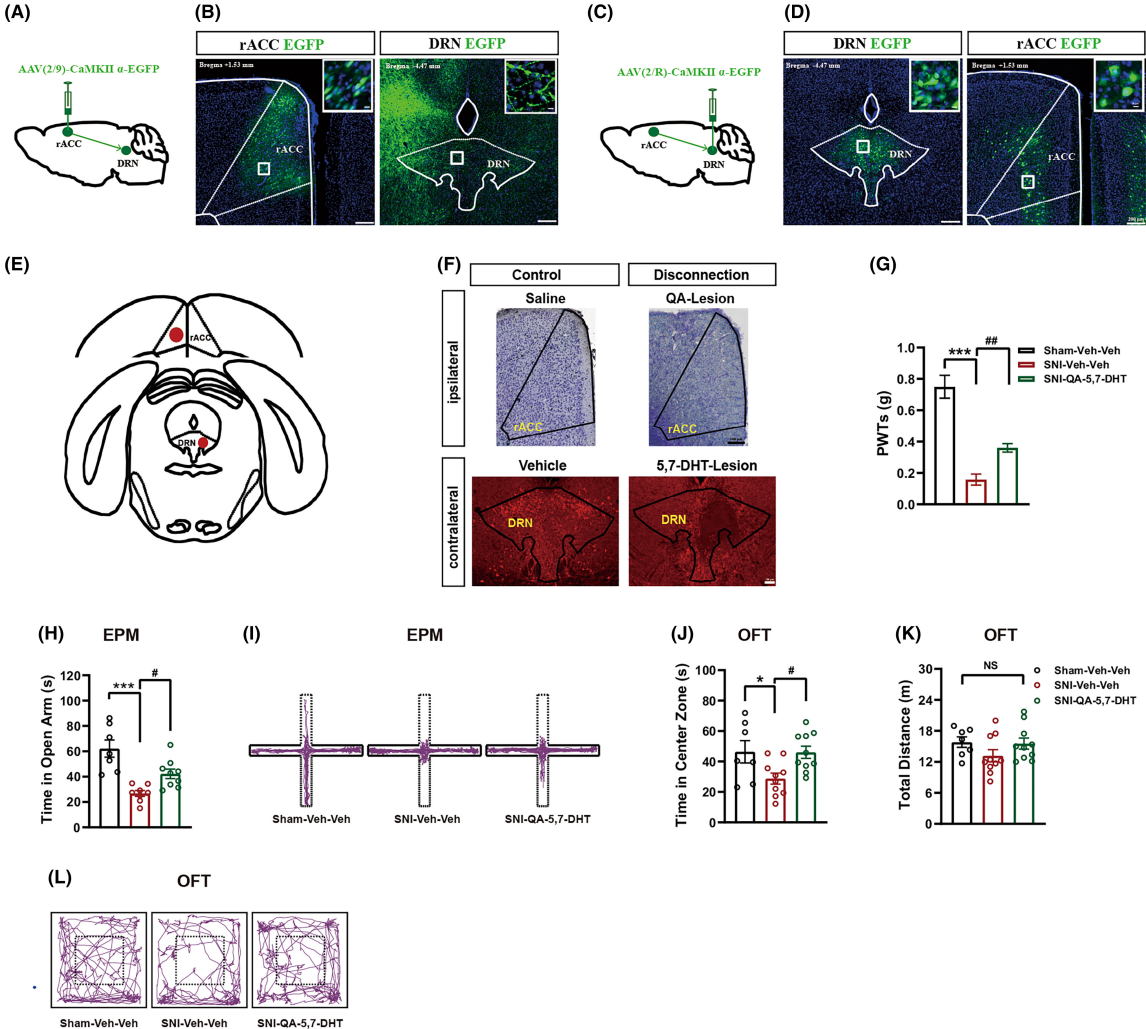
Disconnection of rACC and DRN projections attenuated mechanical allodynia and anxiety‐like behaviors in SNI mice. (A–D) Dissection of the rACC‐DRN pathway. Schematic of the viral injection for anterograde tracing (A). Representative photomicrograph of viral expression in the rACC (left) and EGFP signals in the DRN (right) (B). Schematic of the viral injection for retrograde monosynaptic tracing (C). Representative photomicrograph of viral expression in the DRN (left) and EGFP signals in the rACC (right) (D). Scale bar, 200 μm; 10 μm for the inserted graph. (E) Schematic illustration of contralateral lesions of the rACC (with quinolinic acid) and DRN (with 5,7‐DHT). (F) Brain sections of the rACC (upper, Nissl staining) and DRN (lower, immunofluorescence) to verify neural lesions. Scale bar, 100 μm. (G) Disconnection of rACC and DRN circuit reduced mechanical allodynia at day 14 after SNI (*n* = 8–12). (H) Disconnecting rACC and DRN increased time in open arms decreased by SNI in the EPM (*n* = 7–9). (I) Representative animal tracks of the three groups in the EPM. (J,K) Disconnection of rACC and DRN projections increased the time in the central zone on the OFT reduced by SNI (J) (*n* = 7–10) without affecting locomotor activity (K). (L) Representative animal tracks of the three groups in the OFT. Data are expressed as the mean ± SEM. *p < 0.05 and ***p < 0.001 compared with sham‐Veh‐Veh mice, ^#^ *p* < 0.05 and ^##^ *p* < 0.01 compared with SNI‐Veh‐Veh mice, NS, not significant.

The prelimbic/cingulate (PrL/Cg) cortices have major glutamatergic and monosynaptic projections to DRN^5‐HT^ neurons.^15^ Viral tracing was applied to verify neural projections from rACC^Glu^ to DRN. CaMKIIα, a marker of glutamatergic neurons in the cortex,^41^ was as a viral promoter to specifically express DREADDs in rACC^Glu^ neurons. As shown in Figure 4A, anterograde tracing viruses (AAV2/9‐CaMKIIα‐EGFP) were injected into the right rACC. Three weeks after viral injection, we observed EGFP^+^ cell bodies in the rACC (Figure 4B, left) and EGFP^+^ fibers in the DRN (Figure 4B, right). Furthermore, retrograde tracing viruses (AAV2/R‐CaMKIIα‐EGFP) were injected into the DRN (Figure 4C). Three weeks later, EGFP^+^ fibers in the DRN (Figure 4D, left) and EGFP^+^ cell bodies in the rACC were observed (Figure 4D, right). These data demonstrate that rACC glutamatergic neurons send afferents to the DRN.

The timeline is shown in Figure 1B. QA is excitotoxic to neurons in the ACC. The selective neurotoxin 5,7‐dihydroxytryptamine (5,7‐DHT) destroys 5‐HT axons and terminals in the DRN.^42^ Representative Nissl staining photomicrographs indicating the location of the lesion in the ipsilateral rACC are shown (Figure 4F, upper), while photomicrographs illustrating serotonergic neuron immunoreactivity (5‐HT‐ir) in the contralateral DRN revealed reduced 5‐HT‐ir‐positive neurons in mice that received an intra‐DRN microinjection of 5,7‐DHT (Figure 4F, lower). The disconnection of the rACC and DRN significantly increased the PWT in SNI mice (Figure 4G). As shown in Figure 4H,J, this disconnection of the rACC and DRN alleviated anxiety‐like behaviors on day 14 and 16 after SNI, as the time in the open arms of the EPM and central zone of the OFT were significantly increased. The total distance traveled did not differ among the groups (Figure 4K). Representative animal tracks in the EPM (Figure 4I) and OFT (Figure 4L) are provided. These results demonstrate that the DRN receives glutamatergic inputs from the rACC. And the disconnection of the rACC and DRN mitigates mechanical hyperalgesia and anxiety‐like behaviors induced by chronic neuropathic pain.

### 3.4 Both activation of the rACC^Glu^‐DRN circuit and EA attenuated mechanical allodynia and anxiety‐like behaviors in SNI mice without a synergistic effect

**FIGURE 5:**
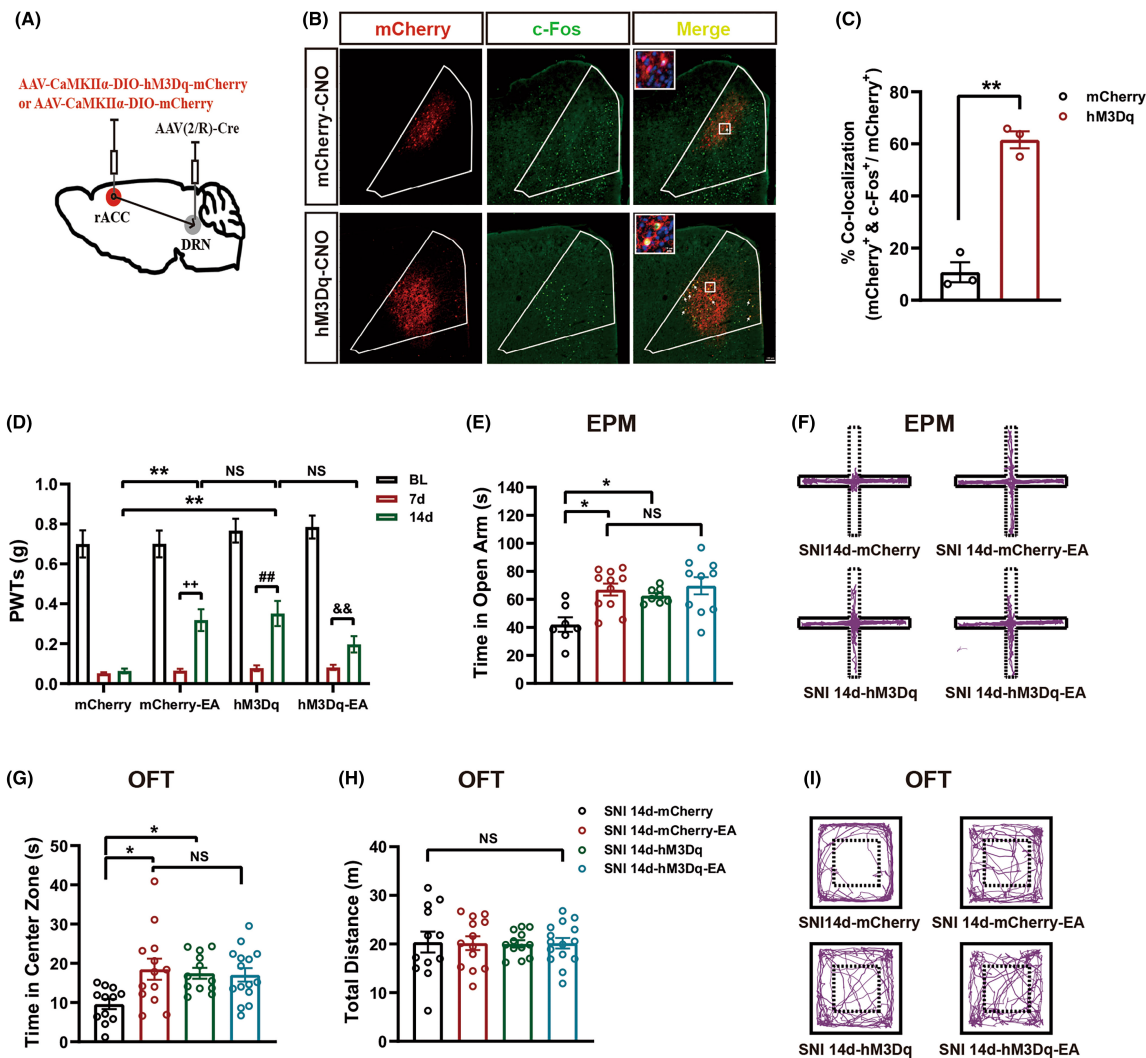
Chemogenetic activation of the rACC^Glu^‐DRN circuit mimicked the therapeutic effects of electroacupuncture (EA), reducing mechanical allodynia and anxiety‐like behaviors in SNI mice, without a synergistic effect. (A) Schematic description for injections of AAV‐CaMKIIα‐DIO‐mCherry or AAV‐CaMKIIα‐DIO‐hM3Dq‐mCherry into the right rACC and AAV2/R‐CaMKIIα‐Cre into the DRN. (B) Representative images showing c‐Fos expression (green) in the rACC transfected with CaMKIIα‐mCherry (upper, red) or CaMKIIα‐hM3Dq‐mCherry (lower, red) after CNO injection in C57BL/6J mice. Arrows indicate c‐Fos expression in CaMKIIα neurons; scale bar, 100 μm, 10 μm for the inserted graph. (C) Proportion of neurons expressing CaMKIIα‐mCherry or CaMKIIα‐hM3Dq‐mCherry that were co‐labeled with c‐Fos in the rACC (*n* = 3, **p < 0.01 compared with mCherry). (D) Time course effect of EA and/or chemogenetic activation of the rACC^Glu^‐DRN circuit on mechanical allodynia in SNI mice (*n* = 10–13). (E) Activation of the rACC^Glu^‐DRN circuit mimicked the effects of EA, reducing the time in the open arms in the EPM (*n* = 7–11). (F) Representative animal tracks of the four groups in the EPM. (G,H) Activation of the rACC^Glu^‐DRN circuit mimicked the effects of EA, reducing the time in the central area in the OFT (G) (*n* = 12–15) in SNI 14d mice with normal locomotor activity (H). (I) Representative animal tracks of the four groups in the OFT. Data are expressed as the mean ± SEM. *p < 0.05 and **p < 0.01 compared with SNI 14d‐mCherry mice on day 14 after SNI, ^++^ *p* < 0.01compared with SNI 14d‐mCherry‐EA mice on day 7 after SNI, ^##^ *p* < 0.01 compared with SNI 14d‐hM3Dq mice on day 7 after SNI, ^&&^ *p* < 0.01 compared with SNI 14d‐hM3Dq‐EA mice on day 7 after SNI, NS, not significant.

To further determine the role of rACC projections to the DRN in mechanical allodynia and anxiety‐like behaviors during chronic pain, we delivered AAV‐CaMKIIα‐DIO‐hM3Dq‐mCherry into the right rACC and AAV(2/R)‐Cre into the DRN, followed by administering intraperitoneal CNO or NS after 21 days (Figure 5A). The timeline is shown in Figure 1C. Specific expression of chemogenetic viruses targeted to CaMKIIα neurons (Figure S1 in Appendix S1). The virus effectively induced mCherry expression in CaMKIIα neurons at the injection site (Figure 5B), and efficient stimulation of hM3Dq‐mCherry‐expressing neurons was confirmed by an increased proportion of CaMKIIα‐mCherry neurons co‐labeled with c‐Fos in the rACC compared with the mCherry group (Figure 5C). Chemogenetic activation of rACC^Glu^‐DRN projection elevates the PWT as well as EA stimulation in SNI 14d mice (Figure 5D), indicating that activation of the projection and EA both exert analgesic effects. In addition, this chemogenetic activation produced anxiolytic effects on the EPM (increased time in the open arms, Figure 5E) and the OFT (increased time in the center zone, Figure 5G), similar to EA. These results indicate that activation of the rACC^Glu^‐DRN circuit mimicked the analgesic and anxiolytic effects of EA under comorbidities of chronic neuropathic pain and anxiety‐like behaviors. EA plus activation of the rACC^Glu^‐DRN circuit increased the PWT (Figure 5D). Moreover, there was no significant difference in mechanical allodynia and anxiety‐like behaviors between mice administered EA plus activation of the rACC^Glu^‐DRN circuit and those treated with either EA or activation of the rACC^Glu^‐DRN circuit (Figure 5D‐I). There was no difference in locomotor activity in the OFT among the groups (Figure 5H). Representative animal tracks in the EPM are shown in Figure 5F, and those in the OFT are shown in Figure 5I. Together, these results suggest that both the activation of the rACC^Glu^‐DRN circuit and EA attenuate SNI‐induced mechanical allodynia and anxiety‐like behaviors. EA plus activation of the rACC^Glu^‐DRN circuit did not produce synergistic effects in terms of relieving mechanical allodynia or anxiety‐like behaviors, indicating that EA may relieve mechanical allodynia and anxiety‐like behaviors in SNI mice by activating the rACC^Glu^‐DRN circuit.

### 3.5 Chemogenetic inhibition of the rACC^Glu^‐DRN circuit did not induce mechanical allodynia and anxiety‐like behaviors in naïve mice

**FIGURE 6:**
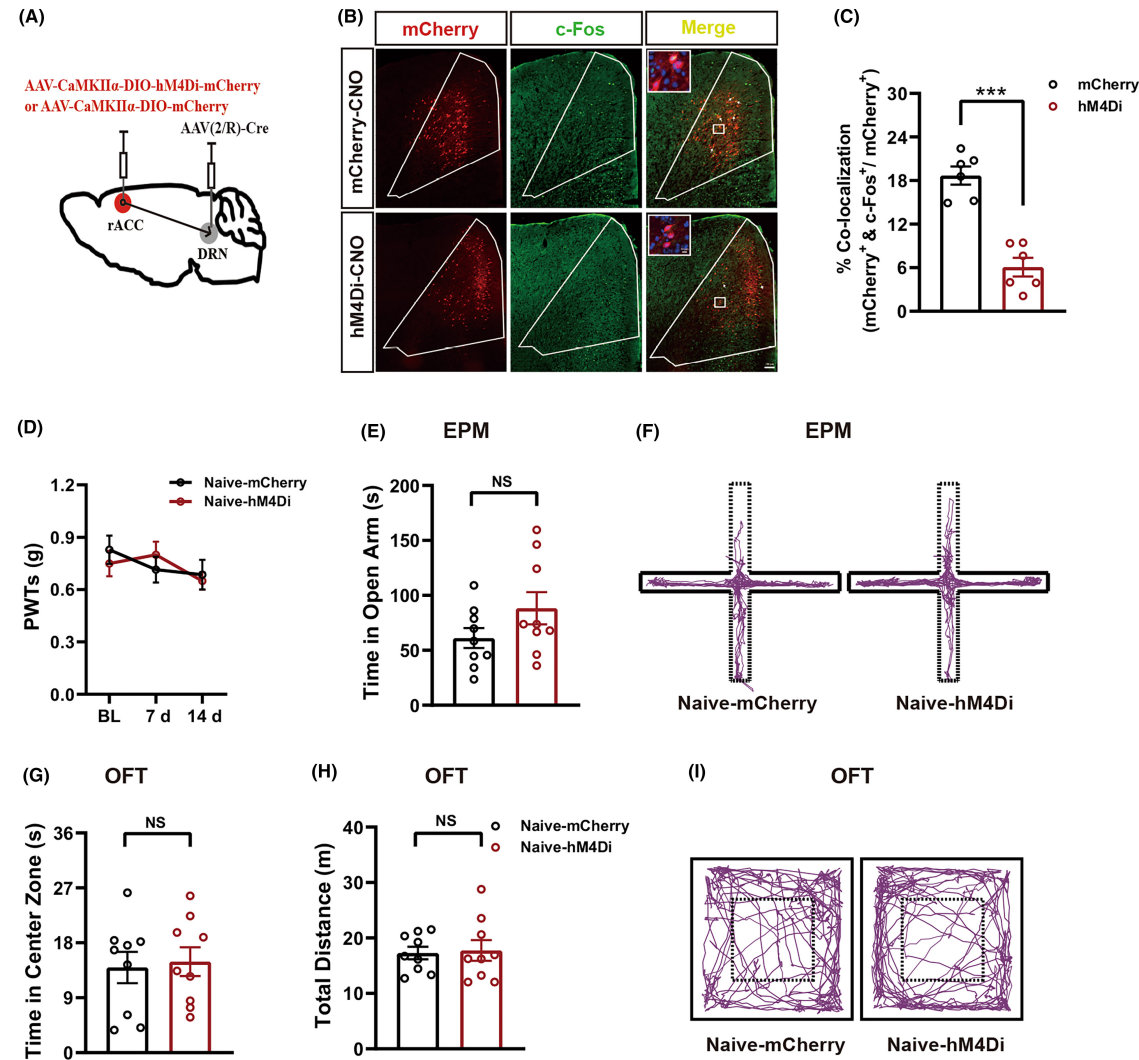
Chemogenetic inhibition of the rACC^Glu^‐DRN circuit did not induce mechanical allodynia and anxiety‐like behaviors in naïve mice. (A) Schematic description of bilateral injections of mCherry or hM4Di‐mCherry into the rACC and AAV2/R‐CaMKIIα‐Cre into the DRN. (B) Representative images showing c‐Fos expression (green) in the rACC transfected with CaMKIIα‐mCherry (upper, red) or CaMKIIα‐hM4Di‐mCherry (lower, red) after CNO injection. Arrows indicate c‐Fos expression in CaMKIIα^+^ neurons; scale bar, 100 μm, 10 μm for the inserted graph. (C) Proportion of neurons expressing mCherry that co‐labeled with c‐Fos in the rACC (*n* = 6 including bilateral brain slices from three mice). (D–I) Specific inhibition of rACC‐DRN inputs in naïve mice did not induce mechanical allodynia (D) (*n* = 9) and anxiety‐like behaviors (E–I). Specific inhibition of rACC^Glu^‐DRN inputs in naïve mice did not change the time in the open arms in the EPM (E) (*n* = 9). The time spent in the central area in the OFT (G) (*n* = 9) was not changed, and mice exhibited normal locomotor activity (H). Representative animal tracks of the two groups in the EPM (F) and in the OFT (I). Data are expressed as the mean ± SEM. ***p < 0.001 compared with mCherry. NS, not significant.

As the activities of rACC^Glu^ and DRN^5‐HT^ neurons were decreased by SNI, we next examined whether chemogenetic inhibition of rACC neurons projecting to the DRN could induce mechanical allodynia and anxiety‐like behaviors in naïve mice. The timeline is shown in Figure 1D. As shown in Figure 6A, we delivered AAV‐CaMKIIα‐DIO‐hM4Di‐mCherry into the bilateral rACC and AAV(2/R)‐Cre into the DRN. Three weeks after viral injection, efficient inhibition of hM4Di‐expressing neurons by administering intraperitoneal CNO was confirmed by the reduced proportion of neurons expressing CaMKIIα‐mCherry that co‐labeled with c‐Fos in the rACC, compared with the naïve‐mCherry group (Figure 6B,C). As shown in Figure 6D, compared with the naïve‐mCherry group, naïve‐hM4Di mice displayed no significant differences in the PWT. Additionally, naïve mice showed no difference in the time in the open arms of the EPM (Figure 6E) and central area of the OFT (Figure 6G) between these two groups. Chemogenetic manipulation had no significant effects on locomotor activity (Figure 6H). Representative animal tracks in the EPM and OFT are shown in Figure 6F,I, respectively. These results demonstrate that chemogenetic inhibition of the rACC^Glu^‐DRN circuit does not cause mechanical allodynia and anxiety‐like behaviors in normal mice.

### 3.6 EA reversed the anxiety‐like behaviors induced by inhibiting the rACC^Glu^‐DRN circuit in SNI 7d mice

**FIGURE 7:**
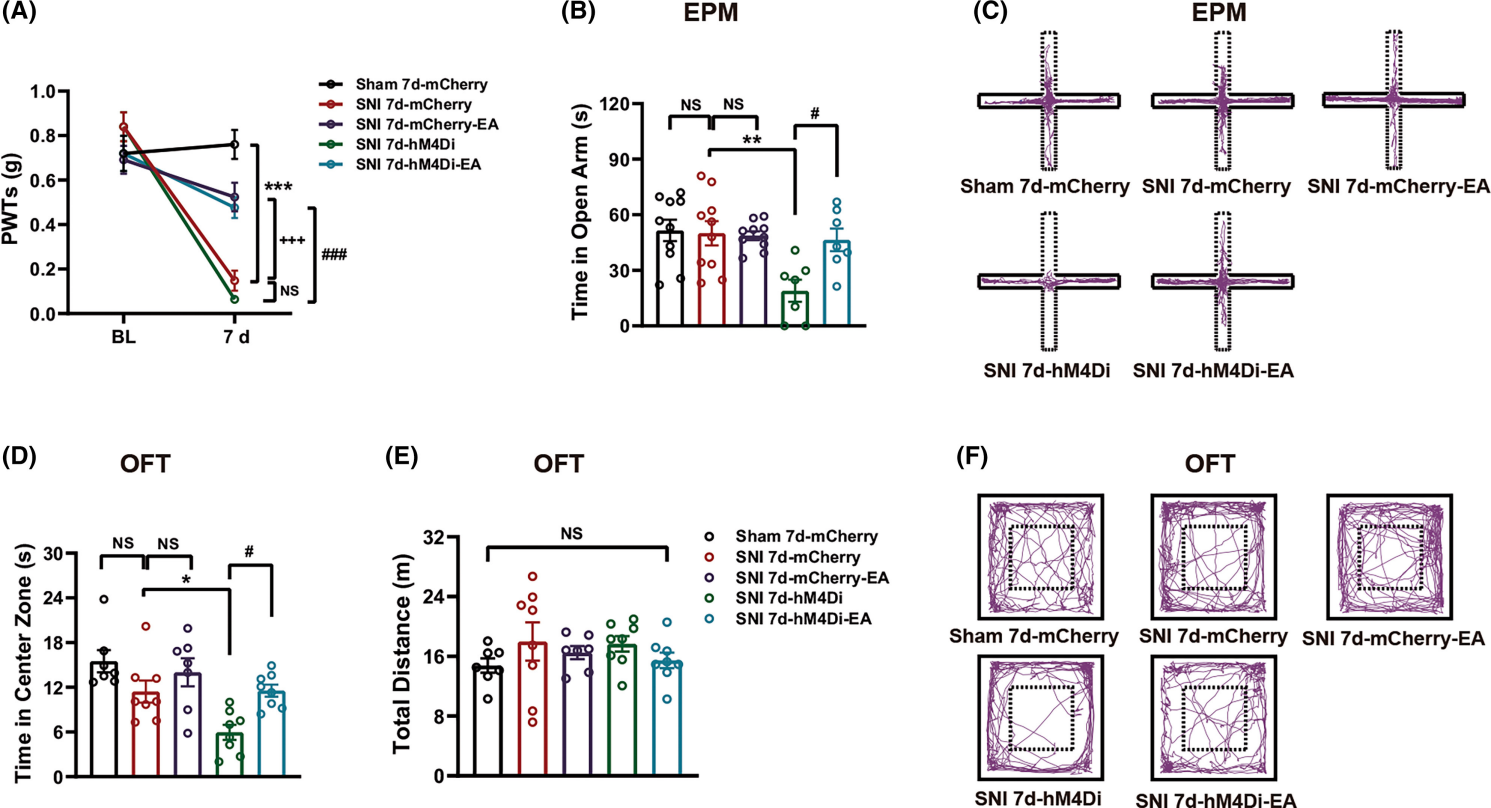
Inhibition of the rACC^Glu^‐DRN circuit evoked anxiety‐like behaviors in SNI 7d mice that were reversed by electroacupuncture (EA). (A) EA attenuated mechanical allodynia induced by SNI (*n* = 7–11). (B) EA blocked the decrease of the time in the open arms induced by inhibition of the rACC^Glu^‐DRN circuit at day 7 post‐surgery in the EPM (*n* = 7–10). (C) Representative animal tracks of the four groups in the EPM. (D,E) EA reversed the decrease of the time in the central area induced by inhibition of the rACC^Glu^‐DRN circuit at day 9 post‐surgery in the OFT (D) (*n* = 7–10) without influencing locomotor activity (E). (F) Representative animal tracks of the four groups in the OFT. Data are expressed as the mean ± SEM. *p < 0.05, **p < 0.01 and ***p < 0.001 compared with sham 7d‐mCherry mice, ^+++^ *p* < 0.001 compared with SNI 7d‐mCherry mice, ^#^ *p* < 0.01 and ^###^ *p* < 0.001 compared with SNI 7d‐hM4Di mice, NS, not significant.

Subsequently, as SNI mice did not show anxiety‐like behaviors at day 7 post‐surgery (SNI 7d; Figure S2C‐G in Appendix S1), we investigated whether inhibition of the rACC^Glu^‐DRN circuit could induce anxiety‐like behaviors in SNI 7d mice. Thus, we used the virus strategy described above. The timeline is shown in Figure 1E. As shown in Figure 7A, SNI 7d‐mCherry mice exhibited mechanical allodynia. Compared with the SNI 7d‐mCherry group, SNI 7d‐mCherry‐EA mice showed an increased PWT and SNI 7d‐hM4Di mice displayed a trend to lower PWT. Interestingly, there was no difference among sham 7d‐mCherry, SNI 7d‐mCherry, and SNI 7d‐mCherry‐EA group, while anxiety‐like behaviors were observed in SNI 7d‐hM4Di mice compared with SNI 7d‐mCherry mice (Figure 7B,D). Furthermore, we examined the effect of EA on mechanical allodynia and anxiety‐like behaviors in SNI 7d‐hM4Di mice. Compared with mice in the SNI 7d‐hM4Di group, the PWT of SNI 7d‐hM4Di‐EA mice was increased (Figure 7A), and the mice treated with EA spent more time in the open arms of the EPM (Figure 7B) and central area of the OFT (Figure 7D), with normal locomotor activity in the OFT (Figure 7E). Representative animal tracks in the EPM are shown in Figure 7C, and those in the OFT are shown in Figure 7F. Taken together, these findings suggest that chemogenetic inhibition of rACC‐DRN circuit contributes to anxiety‐like behaviors in SNI 7d mice (i.e., exerted an anxious promotor) and EA reverses this effect. However, the effect of this chemogenetic inhibition on mechanical allodynia still needs further investigation.

### 3.7 EA relieved mechanical allodynia and anxiety‐like behaviors in SNI mice through the rACC^Glu^‐DRN circuit

**FIGURE 8:**
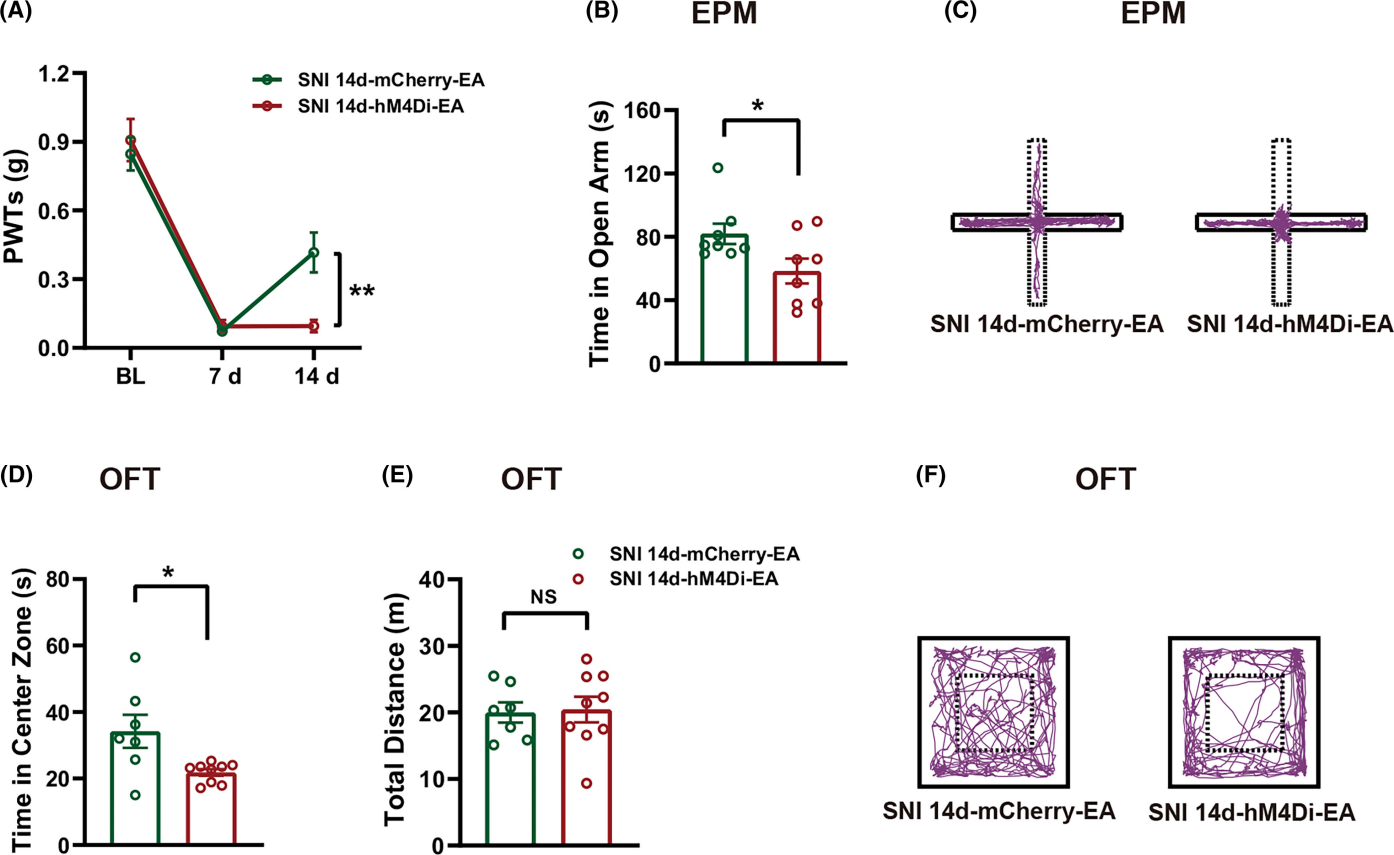
Chemogenetic inhibition of the rACC^Glu^‐DRN circuit blocked electroacupuncture (EA)‐induced reductions in mechanical allodynia and anxiety‐like behaviors in SNI mice. (A) EA did not induce analgesic effect after inhibition of the rACC^Glu^‐DRN circuit (*n* = 13). (B) EA did not increase the time in open arms of the EPM after inhibition of the rACC^Glu^‐DRN circuit at day 14 post‐surgery (*n* = 8). (C) Representative animal tracks of the two groups in the EPM. (D‐E) EA did not increase the time in the central area of the OFT after inhibition of the rACC^Glu^‐DRN circuit at day 16 post‐surgery (D) (*n* = 7‐9) without altering locomotor activity (E). (F) Representative animal tracks of the two groups in the OFT. Data are expressed as the mean ± SEM. *p < 0.05 and **p < 0.01 compared with SNI 14d‐hM4Di‐EA mice; NS, not significant.

To verify the role of the rACC^Glu^‐DRN circuit in EA‐induced alleviation of mechanical allodynia, EA was administered after chemogenetic inhibition of rACC^Glu^‐DRN once every other day (starting on day 8 after SNI). The timeline is shown in Figure 1C. As shown in Figure 8A, compared with SNI 14d‐hM4Di‐EA mice, SNI 14d‐mCherry‐EA mice relieved mechanical allodynia. Additionally, the time spent in the open arms of the EPM (Figure 8B) and central area of the OFT (Figure 8D) in SNI 14d‐mCherry‐EA mice were more than that in SNI 14d‐hM4Di‐EA mice. There was no significant difference in the total distance traveled in the OFT between two groups (Figure 8E). Representative animal tracks in the EPM and OFT are shown in Figure 8C,F, respectively. The results indicate that inhibiting the rACC^Glu^‐DRN pathway prevents the analgesic and anxiolytic effects of EA under pathological conditions.

## 4 DISCUSSION

Under the common mouse model of chronic neuropathic pain induced by SNI, mice begin to exhibit anxiety‐like behaviors on day 12 after SNI^43^; indeed, most research^25^, ^44^ has focused on anxiety‐like behaviors in SNI mice on day 14 post‐surgery. Consistent with these findings, in the present study, we demonstrated that anxiety‐like behaviors (assessed in the EPM and OFT) were observed on day 14 post‐surgery, while no anxiety‐like behaviors were observed on day 7 post‐surgery (Figure S2 in Appendix S1).^45^

Substantial evidence has indicated that common treatment strategies alleviate both chronic pain and affective disorders, suggesting some shared mechanisms and structures.^46^ Among these structures, the rACC and DRN have been separately identified as participating in neural circuits related to the comorbidity model of chronic pain and depression/anxiety.^7^, ^8^, ^12^, ^14^ In this study, we found a decrease in the activities of neurons in the rACC and DRN under chronic neuropathic pain comorbid with anxiety.

The rACC is involved in the affective processing of pain.^47^ Recently, the role of the rACC in chronic pain‐related aversion and anxiety has been clarified. We found that rACC lesions alleviated anxiety‐like behaviors in SNI mice without affecting pain hypersensitivity (Figure S3 in Appendix S1). This result suggests that the rACC participates in anxiety‐like behaviors rather than pain sensory in SNI mice. Previous studies have found that inhibition of the rACC^Glu^‐ventrolateral periaqueductal gray (vlPAG)^8^ and rACC^Glu^‐thalamus^7^ pathways both reduced anxiety‐like behaviors under chronic pain; unexpectedly, we found that the activity of CaMKIIα neurons was reduced under chronic neuropathic pain with anxiety‐like behaviors, which is at odds with prevailing views regarding pain‐related anxiety driven by glutamatergic hyperexcitability within the ACC.^48^ Activity in specific rACC input‐output pathways, rather than the overall level of rACC activity, underlies the contribution of the rACC to chronic pain‐related affective disorders, as Meda^49^ described.

Moreover, a study demonstrated that glutamatergic neurons in the PrL/Cg cortices send biased input to DRN^5‐HT^ neurons.^15^ Our study showed that the DRN receives glutamatergic inputs from rACC neurons. This suggests that the rACC and DRN may constitute a neural circuit that processes chronic neuropathic pain comorbid with anxiety. The precise functional connectivity between the rACC and DRN as well as the role of this circuit in controlling chronic neuropathic pain comorbid with anxiety have not been described.

We also found that disconnection of rACC and DRN projections attenuated mechanical allodynia and anxiety‐like behaviors in SNI mice. Surprisingly, chemogenetic inhibition of the rACC^Glu^‐DRN circuit did not induce mechanical allodynia and anxiety‐like behaviors in naïve mice; instead, it evoked anxiety‐like behaviors in SNI 7d mice without exacerbating hyperalgesia. At day 7 after SNI, the PWT might be at its minimum value; thus, inhibition of the rACC^Glu^‐DRN circuit could have been unable to promote further reduction in the PWT. These findings indicate that the rACC^Glu^‐DRN circuit may play an essential role in processing affective symptoms resulting from chronic neuropathic pain. Interestingly, activation of the rACC^Glu^‐DRN circuit produced an analgesic effect in SNI mice while reducing anxiety‐like behaviors. These results indicate that the rACC^Glu^‐DRN circuit may involve in regulating pain sensation when neuropathic pain occurs accompanied by anxiety‐like behaviors. To further investigate the role of this circuit in regulating perceptions of chronic neuropathic pain, studies need to explore the analgesic effect of inhibiting the circuit in SNI mice with anxiety‐like behaviors. In the present study, we have identified a novel neural circuit for pain modulation.

Previous studies have demonstrated that the rACC, an ascending pathway in a specific circuit, may be involved in regulating both sensory and affective dimensions of pain through connections with different brain regions.^8^, ^50^ As a descending pathway of rACC^Glu^‐DRN circuit, DRN may be involved in the regulation of both mechanical allodynia and anxiety‐like behaviors under chronic neuropathic pain. The involvement of the DRN in neuropathic pain is associated with the 5‐HTergic system.^13^, ^51^ DRN^5‐HT^ neurons not only play a role in the pathogenesis of depression and anxiety but also participate in pain processing. The firing rate of 5‐HTergic neurons is increased in SNI 14d rats compared with sham rats and SNI 7d rats, indicating that the activity of 5‐HTergic neurons is altered by nerve injury in a time‐dependent manner, as expected in a chronic pain condition.^11^ A reduction and/or irregularity in the activity of 5‐HTergic neurons has been linked to the emergence of mood disorders under chronic pain.^12^, ^13^ In this study, we found that the activity of DRN^5‐HT^ neurons was not altered in SNI 7d mice (Figure S2 in Appendix S1), but was reduced in SNI 14d mice accompanied by anxiety‐like behaviors. These data suggest that DRN^5‐HT^ neurons contribute to anxiety‐like behaviors in SNI mice. At present, serotonin‐norepinephrine reuptake inhibitors are one of the recommended first‐line treatments for neuropathic pain.^1^ As 5‐HTergic neurons are more likely to receive synaptic inputs from the anterior neocortex than GABAergic neurons,^15^ 5‐HTergic neurons may function as downstream of the rACC^Glu^‐DRN circuit to regulate both mechanical allodynia and anxiety‐like behaviors. However, to date, there is no direct evidence showing that 5‐HTergic neurons are involved in the rACC^Glu^‐DRN circuit in this study. Hence, we will focus on the role of 5‐HTergic neurons in this circuit in the future.

In this study, we found that 100‐Hz EA produced robust analgesic and anxiolytic effects in SNI mice, as previously reported.^25^ EA inhibits the spontaneous pain‐induced affective response by activating mu opioid receptors in the rACC.^52^ EA alleviates the pain‐depression dyad and upregulates 5‐HT receptors in the DRN of reserpine‐injected rats.^26^ EA regulates anxiety‐like behaviors in CFA rats through the rACC‐thalamus circuit,^7^ while chemogenetic activation of the rACC^Glu^‐vlPAG circuit effectively blocked the analgesic effect of EA in SNI mice without affecting negative emotions.^8^ Thus, EA may play different roles (only analgesic, only anxiolytic, or both analgesic and anxiolytic) in comorbidity of chronic pain and negative emotion by regulating different neural circuits. The complete mechanisms underlying both the analgesic and anxiolytic effects of EA in comorbid chronic neuropathic pain and anxiety‐like behaviors are yet to be fully elucidated. To date, few studies have focused on the role of EA in regulating anxiety‐like behaviors through the rACC or DRN in SNI mice. The present study demonstrated that EA significantly activated rACC^Glu^ and DRN^5‐HT^ neurons under comorbid chronic neuropathic pain and anxiety‐like behaviors. Furthermore, behavioral tests indicated that chemogenetic inhibition of the rACC^Glu^‐DRN circuit in SNI 7d mice mimicked anxiety‐like behaviors at day 14 post‐surgery; these behavioral effects were reversed by EA. Additionally, inhibition of the rACC^Glu^‐DRN circuit blocked the analgesic and anxiolytic effects of EA under chronic neuropathic pain accompanied by anxiety‐like behaviors. Inhibition of the circuit in early phase promotes anxiety, while this inhibition in late phase impedes the effects of EA treatment. Chemogenetic activation of the rACC^Glu^‐DRN circuit produced analgesic and anxiolytic effects in SNI mice, a phenomenon similar to the use of EA. Moreover, we found that EA combined with activation of the rACC^Glu^‐DRN circuit did not exert a synergistic effect, indicating that EA might reduce mechanical allodynia and anxiety‐like behaviors by activating the rACC^Glu^‐DRN circuit. The present study, thus, demonstrates that EA exerts analgesic and anxiolytic effects in mice under chronic neuropathic pain accompanied by anxiety‐like behaviors via activating the rACC^Glu^‐DRN circuit.

In this study, only male adult mice were used to explore the role of rACC^Glu^‐DRN circuit in the comorbidity of chronic neuropathic pain and anxiety. This study was not conducted on female mice and elderly mice, which was the limitation of the study. The prevalence of chronic pain is higher in women than in men and increases significantly with age. The sex and age could have a significant effect on the pain responses, the anxiety‐like behaviors, and the underlying mechanisms. Thus, whether EA exerts analgesic and anxiolytic effects through rACC^Glu^‐DRN circuit under chronic neuropathic pain in female mice and elderly mice needs to be further studied.

## 5 CONCLUSION

In conclusion, our present data suggest that a novel rACC^Glu^‐DRN circuit plays an important role in regulating neuropathic pain as neuropathy develops. Chemogenetic inhibition of the rACC^Glu^‐DRN circuit does not induce mechanical allodynia and anxiety‐like behaviors in naïve mice but evoked anxiety‐like behaviors at early phase of SNI, and activating this circuit produces analgesic and anxiolytic effects at late phase of SNI with anxiety‐like behaviors. The activity of DRN^5‐HT^ neurons is reduced when anxiety‐like behaviors appear during the progression of chronic neuropathic pain. EA exerts analgesic and anxiolytic effects by activating the rACC^Glu^‐DRN circuit, which may have led to the activation of DRN^5‐HT^ neurons. These findings extend current understanding of the neural mechanisms underlying EA's effect on cases of chronic neuropathic pain with anxiety.

## AUTHOR CONTRIBUTIONS

Jianqiao Fang, Xiaomei Shao, and Zui Shen contributed to the study conception and design. Material preparation was performed by Yuerong Chen, Yichen Zhu, Siqi Xiao, Yifang Wang, Chi Zhang, Zenmin Wu, Xiaofen He, and Boyu Liu. Data collection and analysis were performed by Yingling Xu, Xixiao Zhu, Yeqing Chen, and Mengwei Wu. The first draft of the manuscript was written by Yingling Xu, Jianqiao Fang, and Xiaomei Shao. All authors commented on previous versions of the manuscript. All authors read and approved the final manuscript.

## CONFLICT OF INTEREST STATEMENT

The authors declare that they have no conflict of interest.

## Supporting information


Appendix S1

## Data Availability

The data that support the findings of this study are available from the corresponding author upon reasonable request.
