# Supplementary material for: Electroacupuncture alleviates mechanical allodynia and anxiety‐like behaviors induced by chronic neuropathic pain via regulating rostral anterior cingulate cortex‐dorsal raphe nucleus neural circuit
Source: CNS Neurosci Ther. 2023 Jul 3;29(12):4043–58. doi: 10.1111/cns.14328 (PMC10651964; doi:10.1111/cns.14328)
Supplement: Supplementary file 1 — Appendix S1 [file CNS-29-4043-s001.docx]

**Supplementary data**

**Supplementary table 1. Animals were used in the various experimental designs.**

| Experimental designs | Figure | Sample size | Group | | | Number of mice for behavioral tests | | |
| --- | --- | --- | --- | --- | --- | --- | --- | --- |
|  |  |  | Name | SNI? | EA? | PWT | EPM | OFT |
| Effects of EA on mechanical allodynia and anxiety-like behaviors in SNI mice | 2 | 33-41 | Sham | No | No | n=10 | n=8 | n=9 |
|  |  |  | SNI | Yes | No | n=10 | n=8 | n=8 |
|  |  |  | SNI-EA | Yes | Yes | n=11 | n=10 | n=11 |
|  |  |  | SNI-Sham EA | Yes | No | n=10 | n=7 | n=9 |
| Disconnection of rACC and DRN projections attenuated mechanical allodynia and anxiety-like behaviors in SNI mice | 3 | 24-32 | sham-Veh-Veh | No | No | n=8 | n=7 | n=7 |
|  |  |  | SNI-Veh-Veh | Yes | No | n=12 | n=8 | n=10 |
|  |  |  | SNI-QA-5,7-DHT | Yes | No | n=12 | n=9 | n=10 |
| Both activation of the rACC^Glu^-DRN circuit and EA attenuated mechanical allodynia and anxiety-like behaviors in SNI mice | 4 | 36-52 | SNI 14d-mCherry | Yes | No | n=10 | n=7 | n=12 |
|  |  |  | SNI 14d-mCherry-EA | Yes | Yes | n=12 | n=11 | n=13 |
|  |  |  | SNI 14d-hM3Dq | Yes | No | n=12 | n=8 | n=12 |
|  |  |  | SNI 14d-hM3Dq-EA | Yes | Yes | n=13 | n=10 | n=15 |
| Inhibition of the rACC^Glu^-DRN circuit did not induce mechanical allodynia and anxiety-like behaviors in naïve mice | 5 | 15-18 | Naïve-mCherry | No | No | n=7 | n=9 | n=9 |
|  |  |  | Naïve-hM4Di | No | No | n=8 | n=9 | n=9 |
| Inhibition of the rACC^Glu^-DRN circuit evoked anxiety-like behaviors in SNI 7d mice that were reversed by EA | 6 | 38-51 | Sham 7d-mCherry | No | No | n=10 | n=10 | n=7 |
|  |  |  | SNI 7d-mCherry | Yes | No | n=10 | n=10 | n=8 |
|  |  |  | SNI 7d-mCherry-EA | Yes | Yes | n=11 | n=10 | n=7 |
|  |  |  | SNI 7d-hM4Di | Yes | No | n=10 | n=7 | n=8 |
|  |  |  | SNI 7d-hM4Di-EA | Yes | Yes | n=10 | n=7 | n=8 |
| Inhibition of the rACC^Glu^-DRN circuit blocked EA-induced reduction in mechanical allodynia and anxiety-like behaviors in SNI mice | 7 | 16-26 | SNI 14d-mCherry-EA | Yes | Yes | n=13 | n=8 | n=7 |
|  |  |  | SNI 14d-hM4Di-EA | Yes | Yes | n=13 | n=8 | n=9 |

**Supplementary figure 1. Chemogenetic viruses were expressed mainly in rACC^Glu^ neurons**

We used chemogenetic manipulation to explore the role of the rACC^Glu^-DRN circuit in comorbidity of chronic neuropathic pain and anxiety. To directly label the rACC^Glu^ projections to the DRN, we delivered AAV-CaMKIIα-DIO-mCherry into the rACC and AAV(2/R)-Cre into the DRN (Figure S1A, left). The virus effectively induced mCherry expression in neurons at the injection site (Figure S1A, right). Representative immunohistochemistry photomicrographs displaying mCherry signal co-localization with CaMKIIα in the rACC (Figure S1B). As shown in Figure S1C, more than 82% of mCherry-labeled neurons expressed CaMKIIα in mice. These results indicate the viability of the virus strategy.


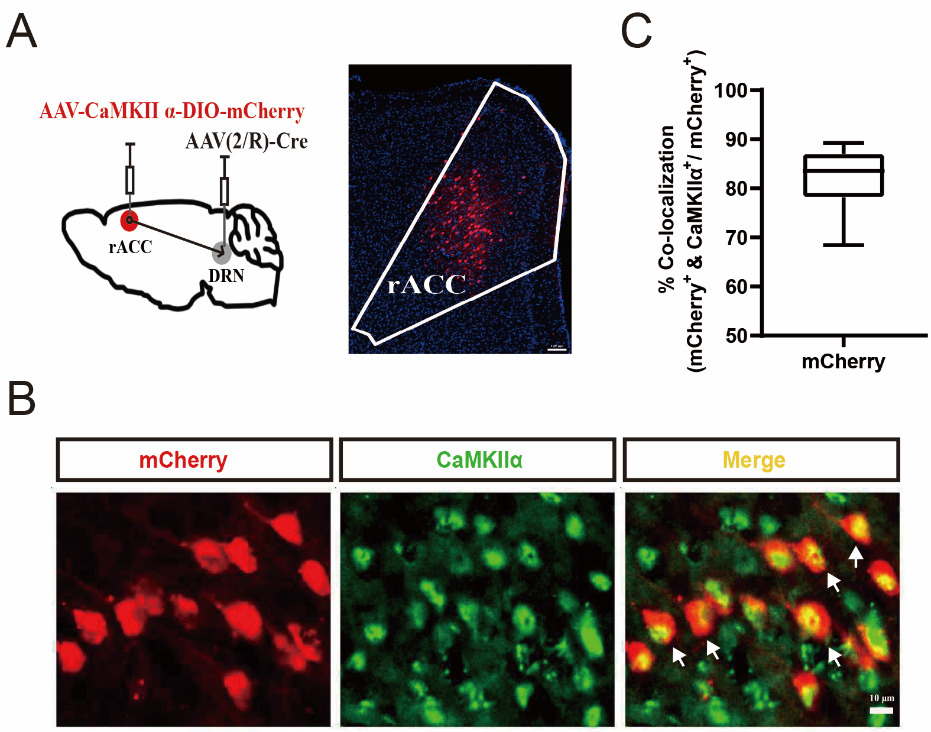


**Supplementary** **figure 1. Specific expression of chemogenetic viruses targeted to glutamatergic neurons.**

(A) Schematic diagram of bilateral injections of AAV-CaMKIIα-DIO-mCherry into the rACC and AAV(2/R)-CaMKIIα-Cre into the DRN (left) and typical photomicrograph of viral expression within the rACC (right). Scale bar, 100 μm. (B) mCherry signals (red) were colocalized with the glutamatergic neuronal marker CaMKIIα (green) in the rACC. Scale bar, 10 μm. (C) Percentage of mCherry-labeled neurons that expressed CaMKIIα in the rACC (n=15 slices from 3 mice).

**Supplementary figure 2. Mice did not show anxiety-like behaviors on day 7 post-SNI and the activity of 5-HTergic neurons in the DRN remained unchanged**

We wondered whether SNI 7d mice would display anxiety-like behaviors and how the activity of DRN^5-HT^ neurons changed over time. The experimental design is shown in Figure S2A. Although the SNI 7d mice exhibited a significant decrease in the PWT (Figure S2B), anxiety-like behaviors did not appear at day 7 post-surgery (Figure S2C, E) with normal locomotor activity (Figure S2F). Representative animal tracks in the EPM (Figure S2D) and OFT (Figure S2G) are provided. Subsequently, we explored the activity of DRN^5-HT^ neurons during chronic neuropathic pain without anxiety. Representative images of 5-HT (red) co-stained with c-Fos (green) in the DRN at day 9 post-surgery are shown in Figure S1H. Immunostaining indicated that the numbers of c-Fos-positive neurons (Figure S2I) and total 5-HTergic neurons (Figure S2J) did not differ between these two groups, nor did the number of 5-HT-expressing neurons co-labeled with c-Fos in the DRN (Figure S2K). Together, the above experiments demonstrate that SNI mice do not show anxiety-like behaviors at day 7 post-surgery with unchanged activity of DRN^5-HT^ neurons.

**
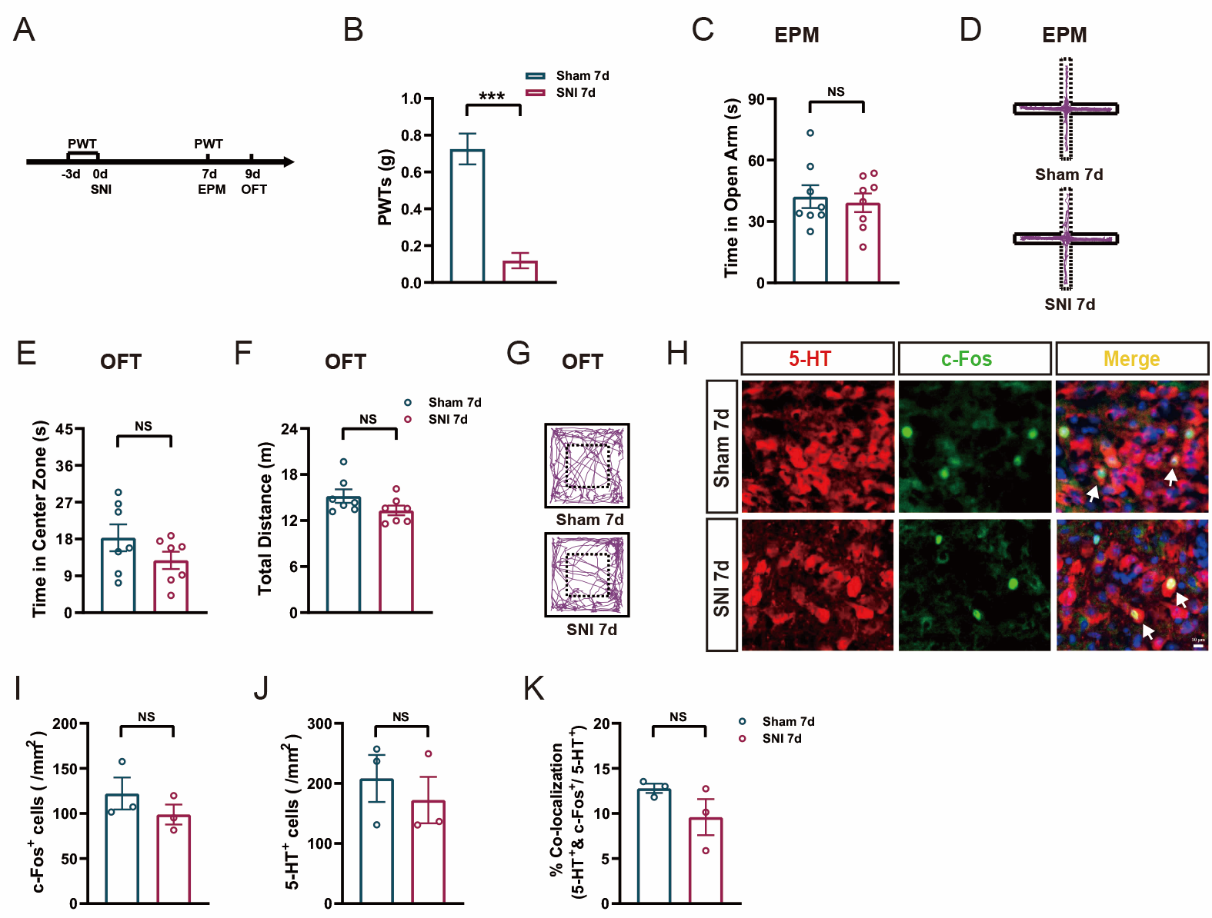
**

**Supplementary figure 2.** **SNI 7d mice exhibited** **mechanical allodynia but not anxiety-like behaviors or altered activity of DRN^5-HT^ neurons.**

(A) Timeline of SNI surgery and behavioral testing. (B) Mechanical allodynia was increased in SNI 7d mice (n=8). (C) The time in the open arms in the EPM did not differ between the two groups (n=8). (D) Representative animal tracks in the EPM. (E-F) The time in the center area in the OFT did not differ between the two groups (E) (n=7), nor did locomotor activity (F) (n=7). (G) Representative animal tracks in the OFT. (H) Representative images indicating that 5-HT (red) was co-stained with c-Fos (green) in the DRN of the two groups. Arrows indicate c-Fos expression in 5-HT^+^ neurons; scale bar, 10 μm. (I) The number of c-Fos-positive neurons in the DRN remained unchanged (n=3). (J) The number of 5-HT-positive neurons in the DRN remained unchanged (n=3). (K) Proportion of neurons expressing 5-HT that co-labeled with c-Fos in the DRN (n=3). At least five slices were taken from each mouse. Data are expressed as the mean ± SEM. ^***^p<0.001 compared with sham 7d mice, NS, not significant.

**Supplementary figure 3. The rACC was required for anxiety-like behaviors in** **SNI mice.**

The bilateral rACC was lesioned by microinjection of QA (an excitotoxin). The experimental design is shown in Figure S3A. SNI mice injected with QA into the rACC exhibited mechanical allodynia similar to SNI mice injected with saline (Figure S3B); thus, there did not appear to be a significant effect of QA treatment on mechanical allodynia. However, as shown in Figure S3C and S3E, QA treatment alleviated anxiety-like behaviors on day 14 and 16, as the time in the open arms and central area was significantly increased. The total distance traveled did not differ among the groups (Figure S3F). Representative animal tracks in the EPM (Figure S3D) and OFT (Figure S3G) are provided. Taken together, the above experiments demonstrate that the rACC is required for anxiety-like behaviors induced by chronic pain, although it is not responsible for mechanical hyperalgesia.


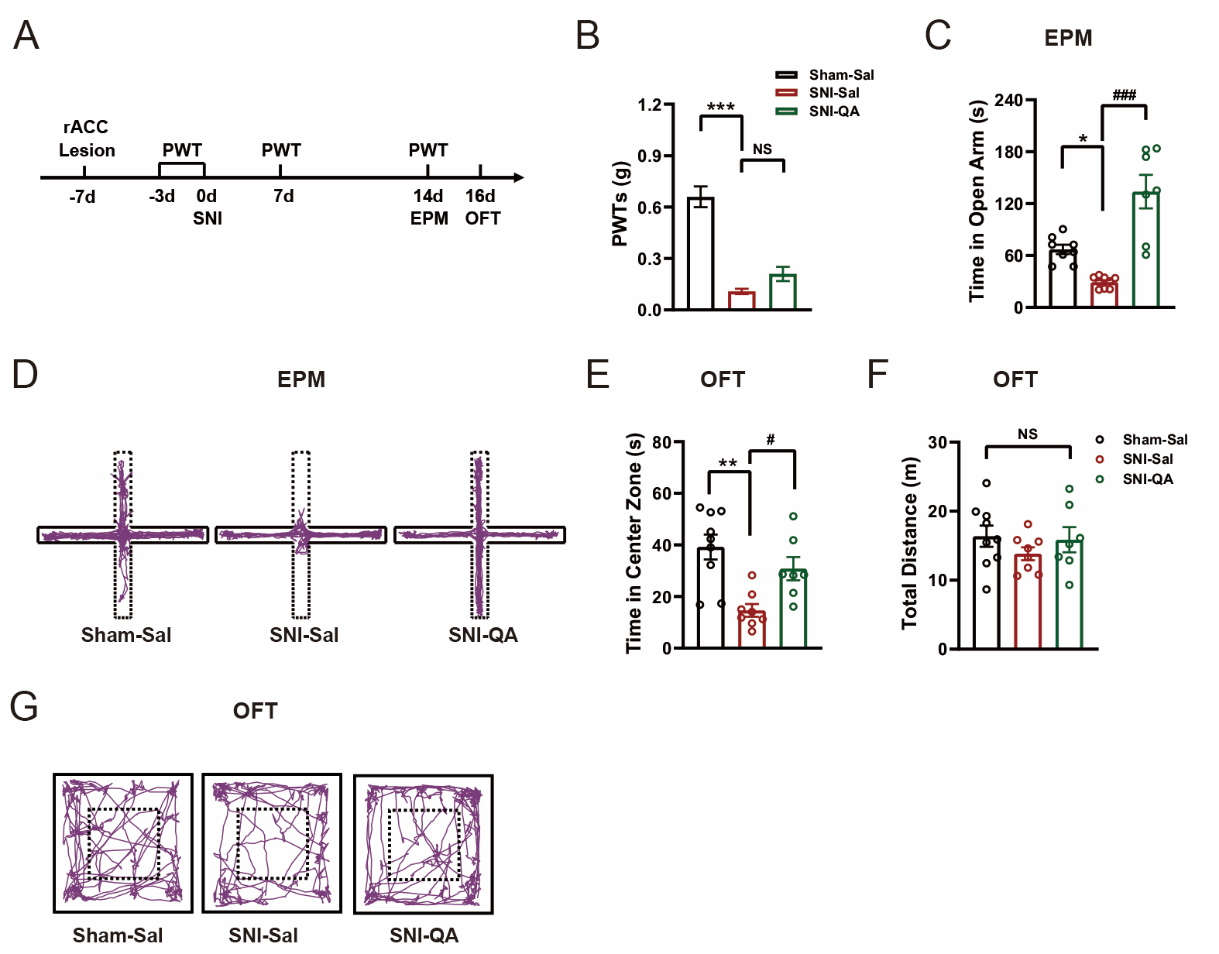


**Supplementary** **figure 3.** **rACC lesions reduced anxiety-like behaviors in SNI mice without affecting mechanical allodynia.**

(A) Timeline of rACC lesions, SNI and behavioral tests. (B) Mechanical hypersensitivity in SNI 14d mice was not altered by rACC lesions (n=10-12). (C) rACC lesions increased the time in the open arms in the EPM (n=7-9). (D) Representative animal tracks from sham-saline (Sal), SNI-Sal and SNI-QA mice in the EPM. (E-F) rACC lesions increased the time in the center area in the OFT (E) (n=7-9) without influencing locomotor activity (F). (G) Representative animal tracks from these groups in the OFT. Data are expressed as the mean ± SEM. ^*^p<0.05, ^**^p<0.01 and ^***^p<0.001 compared with sham-Sal mice, ^#^p<0.05 and ^###^p<0.001 compared with SNI-Sal mice; NS, not significant.
